# Respiratory health effects of e-cigarette substitution for tobacco cigarettes: a systematic review

**DOI:** 10.1186/s12954-023-00877-9

**Published:** 2023-10-04

**Authors:** Maria Ahmed Qureshi, Robin W. M. Vernooij, Giusy Rita Maria La Rosa, Riccardo Polosa, Renee O’Leary

**Affiliations:** 1 Department of Clinical and Experimental Medicine, University of Catania, Catania, Italy; 2 Department of Nephrology and Hypertension, University Medical Center Utrecht and Julius Center for Health Sciences and Primary Care, Utrecht, Netherlands; 3 Centre of Excellence for the Acceleration of Harm Reduction, University of Catania, Via Santa Sofia, 89 Torre Biologica 11 Piano, 95123 Catania, Italy

**Keywords:** Electronic cigarettes, E-cigarettes, ENDS, Respiratory diseases, Lung function, Spirometry, Systematic review

## Abstract

**Background:**

E-cigarettes (electronic nicotine delivery system, ENDS) have been presented as a harm reduction strategy for people who smoke tobacco cigarettes but who cannot achieve abstinence, or for those who wish to continue to enjoy nicotine and the habit of smoking. What are the health effects of the substitution of ENDS for tobacco cigarettes? This systematic review evaluates the evidence of human clinical tests on the respiratory effects of ENDS use in participants who smoke tobacco cigarettes.

**Methods:**

A registered and published protocol was developed conforming to PRISMA 2020 and AMSTAR2 standards. The literature search was conducted in PubMed, Scopus, and the CENTRAL Cochrane Library and updated to May 2022. Three supplementary searches and a grey literature search were performed. Studies were evaluated with the JBI quality tools and the Oxford *Catalogue of Bias*. Due to the heterogeneity (diversity) of the studies, a narrative data synthesis was performed on the test findings plus three sub-group analyses.

**Results:**

The review consists of sixteen studies and twenty publications. Spirometry tests comprised the majority of the data. In total, 66 respiratory test measurements were reported, out of which 43 (65%) were not significant. Statistically significant findings were mixed, with 9 tests showing improvements and 14 measuring declines, none of which was clinically relevant. Ten studies were rated at a high risk of bias, and six had some concerns primarily due to inadequate research designs and the conduct of the studies. Reporting bias was documented in thirteen studies.

**Conclusions:**

Most of the studies showed no difference in respiratory parameters. This indicates that ENDS substitution for smoking likely does not result in additional harm to respiratory health. Due to the low quality of the studies, confidence in the conclusions is rated as low. Robust studies with a longer duration and sufficient power are required to validate any potential benefits or possible harms of ENDS substitution.

*Registration* PROSPERO #CRD42021239094, International Registered Report Identifier (IRRID): DERR1-10.2196/29084.

**Supplementary Information:**

The online version contains supplementary material available at 10.1186/s12954-023-00877-9.

## Introduction

Tobacco use annually causes over 8 million deaths and a loss of 150 million disability-adjusted life-years [1]. Smoking is the attributable mortality risk factor for many respiratory diseases [2]. Some researchers claim that e-cigarettes are potentially safer than smoking [3–6] and, therefore, could be a harm-reduction tool. E-cigarettes are called electronic nicotine delivery systems (ENDS); they are also called vapes, vape pens, tanks, mods, pod-mods, and JUUL. A review by the US National Academies of Sciences Engineering and Medicine states, “There is substantial evidence that except for nicotine, under typical conditions of use, exposure to potentially toxic substances from e-cigarettes is significantly lower compared with combustible tobacco cigarettes” [7]. The Royal College of Physicians (UK) and a number of researchers encourage people who smoke to switch from cigarettes or other combustible tobacco products to what they have evaluated as the less toxic and potentially safer ENDS [8–10]. While much research has focused on ENDS as a cessation tool [11], for people who do not wish to quit consuming nicotine, the substitution of ENDS may be a tobacco harm reduction option [12, 13].

To weigh the potential benefits and risks of ENDS substitution for tobacco smoking, we conducted a systematic review to answer the question, “What are the respiratory health effects, both acute and longer-term, resulting from the substitution of ENDS for tobacco cigarettes?” Our systematic review aims to critically assess and synthesize the available human clinical studies on the respiratory health effects of ENDS substitution by people who smoke.

## Methods

Our research question is structured with PICOS framing (Population, Intervention, Comparator, Outcome, Studies) as follows:

- Population: adults who smoke tobacco cigarettes.
- Intervention: substitution of ENDS for cigarettes.
- Comparator: either within-subject changes or comparison to participants who continue to smoke.
- Outcomes: changes in baseline to post-intervention test measurements from spirometry tests (FEV_1_, FVC, FEF_25–75_, PEF, FEV_1_/FVC%), impulse oscillometry, and lung function tests (total lung capacity, residual volume, and expiratory reserve volume).
- Studies: randomized controlled trials, quasi-experimental clinical trials, and longitudinal cohort studies.

This review adheres to the guidelines of the Preferred Reporting Items for Systematic Reviews and Meta-Analysis (PRISMA) 2020 [14]. The protocol for this systematic review has been registered with PROSPERO #CRD42021239094 and has been published in a peer-reviewed journal [15]. The PRISMA 2020 checklist is in Additional file 1: Table S1. Below is an overview of how the review was conducted. A more detailed description of the search and study selection processes is available in the published protocol [15].

### Search strategy

The database search was performed on January 31, 2021, with updates on April 29, 2021, and May 18, 2022. The publication date delimiter for the database search was 2010, and the databases used were Scopus, PUBMED, and the CENTRAL Cochrane Library. The search terms “electronic nicotine” AND “e-cigarette” were combined with OR for the respiratory keywords “respiratory,” “lungs” and “pulmonary.” The search syntaxes are displayed in Additional file 1: Figure S1. Common terms for ENDS (“Juul” “vaping”) were tested as keywords but did not retrieve any additional studies for inclusion. Keyword “vap*” was not used because it yielded thousands of false retrievals of chemistry studies.

Three secondary searches were conducted in February 2021. First, the reference lists of systematic and narrative reviews published since 2018 were examined for additional studies. Next, a secondary literature search was conducted in Google Scholar. Two experts in the field of ENDS research reviewed the list of included studies. Finally, a grey literature search was conducted at the websites of 53 respiratory medical organizations (listed in Additional file 1: Table S2).

### Inclusion, exclusion, and study eligibility criteria

Study designs included in the review were human subject research with randomized and non-randomized controlled trials, clinical trials, prospective and retrospective cohort studies, and case–control studies. The first exclusion of articles was conducted on titles, and where a title was not sufficient for a determination, the abstract was reviewed. In vitro (cell), animal, and cross-sectional studies were excluded.

The second process of inclusion was a full paper review. Three inclusion criteria were applied. One, a study had to be one of the research designs listed above. Two, a study was required to have either a comparator group who smoked tobacco (cigarettes) or within-subject testing of participants who had substituted ENDS for smoking. Third, the study had to report an outcome of a respiratory test. All three criteria had to be satisfied for a study to be included.

The inclusion and exclusion of studies were conducted independently by two reviewers after training, and initial discrepancies were resolved by discussion. Where agreement could not be reached, the Project Leader made the final decision. Inter-rater reliability was 98% for title sorting and 95% for full paper review.

#### Data extraction

The data extraction process was conducted independently by two reviewers after training using a pre-specified data extraction form drawn from the *JBI Manual* [16] and the *Cochrane Collaboration Handbook* [17]. Any discrepancies in data extraction were resolved by discussion.

### Quality assessment and risk of bias

Two independent reviewers assessed the study quality using the JBI quality assessment tools [18] and a report list of biases drawn from the Oxford Centre for Evidence-Based Medicine *Catalogue of Bias* [19] further supplemented with our teams’ prompt questions. In the case where multiple articles were published on one study, each article was assessed separately. Interim publications of longer duration studies were not assessed for quality, but were referenced for data as necessary. Discrepancies were resolved by discussion.

The overall rating of study quality consisted of a combination of the JBI score and the biases report. Studies were rated in three classifications from the Cochrane guidelines [17]: low risk of bias, some concerns of bias, and high risk of bias. The rater (RO) was blinded to study outcomes and funders. The final rating was endorsed by the team members who conducted the JBI and bias assessments. The rating rubric is in Additional file 1: Table S3.

### Data analysis and synthesis

As per protocol, we conducted a narrative synthesis by study design, population, test measurements, and biases.

A meta-analysis was not conducted due to the heterogeneity between the studies. These differences across studies included the ENDS nicotine strength, the ENDS models, wide disparities in study populations, and differing tests.

Three sub-group analyses of testing measurements were conducted for (1) concurrent use of ENDS and cigarettes (dual use), (2) populations with prior disease conditions, and (3) ENDS use of a duration of 1 year or longer.

Sensitivity analyses were conducted. One excluded all studies at high risk of bias. The second was on the effect modifications on findings. Finally, the certainty of the evidence was evaluated with Grading of Recommendations Assessment, Development, and Evaluation (GRADE) [20, 21].

### Deviations from protocol

There were a few deviations from the protocol. Due to journal word count limits and reporting needs, we excluded the narrative summary of individual studies. A sensitivity analysis for conflict of interest for industry-funded studies was not conducted because all industry studies were at high risk of bias (independently of their industry funding). An analysis of effect modifications was added to conform to PRISMA 2020 requirements. Because no meta-analysis was conducted, a formal assessment of publication bias could not be performed. The planned data repository was changed.

As per protocol, we have transitioned the review from a living systematic review (ongoing searches and updates) to a completed systematic review with the final search date of May 2022 because of the insufficient number of new studies published. Only one new article for inclusion was published in the 18 months after the baseline search, so the living component is not justified at this time.

## Results

The search results are reported in the PRISMA 2020 flow diagram, Additional file 1: Figure S1. Publications excluded at full paper review, including “near misses” [14], are listed in Additional file 1: Table S4 with their reason for exclusion.

Our systematic review retrieved sixteen studies [22–37] with twenty publications [38–41]. One of the studies had two publications with different analyses [34, 38] so both were referenced together. Three studies [39–41] were interim publications of longer-term studies [32, 33]; only the final results were included in the analysis. Basic information on the studies and publications are reported in Table 1 Characteristics of studies. The studies were conducted in Greece (5), United Kingdom (4), USA (3), Italy (2), and one each from Belgium and Hungary. The participants ranged in age from 18 to 73, comprising 1,357 participants who smoked. Six studies included participants with asthma or chronic obstructive pulmonary disease (COPD). Seven studies had acute testing data; nine studies presented follow-up data ranging from 5 days to 5 years. Ten studies were rated at high risk of bias, six were rated as some concerns, and zero studies were rated as at low risk of bias, see Table 2.

**Table 1:** Characteristics of studies

Study References Country	Design	Funder	Participants	Intervention	Duration
*Acute*					
Chaumont [23] Belgium	RCT	Private Non-profit	N = 9 randomly selected from a pool of Males 18, Females 7 Age: 23 (± 0.4) Very occasional smoking, less than 1 cigarette a week	25 total puffs, one every 30 s [excessive exposure] non-nicotine	Tested 10 min after vaping
Flouris [27] Greece	Quasi	No funding	Participants who smoke n = 15 Male 8, Female 7 Age: 23.5–54 years “Long-term smoking”	3–14 puffs (average 10) on nicotine ENDS	Tested 1 h after vaping
Kerr [29] UK	RCT	Non-profit	N = 20 male only Age: 31.6 (± 10.5)	15 puffs of nicotine ENDS	Tested immediately after vaping
Kotoulas [30] Greece	RCT	University Hospital	N = 50 *n* = 25 moderate asthma, smoking *n* = 25 “healthy” participants who smoke Asthma: Male 13, Females 12 Asthma age: 40.64 (± 10.81)	10 puffs of nicotine ENDS	Tested 5 min after vaping
Lappas [22] Greece	Quasi	Non-profit	N = 54 *n* = 27 mild asthma, dual use (> 1 cigarette past 30 days) (nicotine ENDS use ≥ 3 months) Age: 18–35	10 puffs on nicotine ENDS	Tested 30 min after vaping
Palmidas [31] Greece	Quasi	Funding NR	N = 76 participants who smoke *n* = 16 COPD *n* = 11 asthma > 15 cigarettes/day	Nicotine or non-nicotine ENDS use for 10 min	Tested 0, 15, and 30 min after vaping
Vardavas [35] Greece	Quasi	Non-profit	N = 30 Male 14, Females 16 Age: 19–56, Mean 34.8	5 min nicotine ENDS	Tested immediately after vaping
*Follow-up*					
Barna [24] Hungary	Quasi	Funding NR	N = 24 male only Age: 20–64 Former heavy smoking (not defined) Current use of ENDS Length of ENDS use: NR	ENDS users reverted to smoking 20–25 cigarettes daily	7 days
Cravo [25] UK	RCT	Industry	n = 286 ENDS n = 101 TC ~ 55% Male Age: 34.1 (± 10.6) TC use: 11–20/day (range 5–30)	Nicotine ENDS supplied TC abstinence requested	12 weeks
D’Ruiz [26] US	RCT	Industry	N = 105 65% Male Age: 37.8 (± 11.1) TC use 18.8 (± 6.5)/day	Clinical confinement Two groups: (1) exclusive nicotine ENDS (2) 50% of usual TC consumption plus nicotine ENDS	5 days
Hickling [28] UK	Quasi	Non-profit Government	N = 50 38 Male, 12 Female Mean age: 38.96 TC use: 17.9/day (mean)	Provided with nicotine ENDS used ad lib	24 weeks
Polosa asthma [33] Italy [39*]	Cohort	Funding NR	N = 16 mild to moderate asthma at follow-up *n* = 10 Exclusive ENDS use Male 7, Female 3 Age: 33.4 (± 11.6) *n* = 6 Dual users Age: 45.7 (± 9.9)	Participants continued to use ENDS or dual use	24 months
Polosa COPD [32] Italy [40*, 41*]	Cohort	University	N = 39 patients with COPD at F/U *n* = 20 COPD ENDS group (dual and exclusive use) Males 17, Females 3 Age: 66.9 (± 5.8)	Ad lib ENDS use	60 months
Pulvers [38]/ Arnold [34] US	RCT	Government	N = 186 at F/U ENDS *n* = 125 TC *n* = 61 Male 111, Female 75 Age: 43.3 (± 12.5) TC use: 12.1 (± 7.2)/day 92 African American 94 Latinx	Nicotine ENDS with education to reduce TC	6 weeks
Veldheer [36] US	RCT	Government	n = 191 participants using ENDS 56.5% female Age: 46.5 (± 11.4) TC use: 18.1 (± 6.5)/day	Nicotine and non-nicotine ENDS ad lib “Encouraged” to reduce TC	3 months
Walele [37] UK	RCT	Industry	N = 102 Males 57, Females 45 Age: 38.7 (± 10.2)	Nicotine ENDS supplied Requested TC abstinence and exclusive use of supplied ENDS	24 months

ENDS, electronic nicotine delivery system, e-cigarette; F/U, follow-up; NR, not reported; Quasi, quasi-experimental study, a non-randomized experimental study testing cause and effect; TC, tobacco cigarette

*Interim publication of study findings

**Table 2:** Risk of bias assessment, JBI assessments and study biases

Study/rating	JBI	JBI blinding items	Other JBI items	Potential sources of research bias	Reporting bias
Barna [24] HIGH RISK OF BIAS	6/9	NA	Dissimilar participants Limited measurements Differences in outcome measurement	Small sample size All participants male Excessive cigarette exposure No wash-out period	Test measurements NR Spin: pro-ENDS conclusion
Chaumont [23] HIGH RISK OF BIAS	8/13	Allocation not concealed No participant blinding No treater blinding	Subgroup for pulmonary tests randomly selected from full participant group Testing only non-nicotine use	Excessive ENDS exposure Very small sample size Participants occasional smokers, short smoking history	Focus on surrogate markers Substantial and multiple discrepancies between data and discussion Spin: over-generalization
Cravo [25] HIGH RISK OF BIAS	9/13	Allocation not concealed No participant blinding No treater blinding Assessor blinding unclear	No additional items	Lung function test conducted only on cohort 1	Spin: NS findings described as causal
D’Ruiz [26] HIGH RISK OF BIAS	6/13	Allocation unclear No participant blinding No treater blinding Assessor blinding unclear	Randomization unclear Treatment groups dissimilar Mixed model statistical basis unclear	Ad libitum use, consumption levels not recorded Potential volunteer bias: high compensation	None noted
Flouris [27] SOME CONCERNS OF BIAS	8/8	NA	No items	Indirectness of ENDS (device design no longer on the market) Participants: wide variation in smoking history	Spin: pro-ENDS conclusion
Hickling [28] HIGH RISK OF BIAS	7/9	NA	No control groups. Follow-up incomplete	Small sample size. Participants > 70% male. No recruitment information	Test measurements NR Spin: emphasis on pro-ENDS results
Kerr [29] HIGH RISK OF BIAS	6/10	No allocation concealment No participant blinding No treater blinding Assessors blinding unclear	No items	Small sample size. Participants all male	Discrepancy between text and Fig. 3a
Kotoulas [30] HIGH RISK OF BIAS	9/12	Allocation not concealed No participant blinding	No randomization	No recruitment information Very high nicotine strength	Spin: secondary tests presented, not health outcomes Spin: over-generalization
Lappas [22] SOME CONCERNS OF BIAS	8/8	NA	No items	Current smoker definition overly broad (≥ 1 cigarette past 30 days)	Spin: focus on secondary endpoints
Palmidas [31] HIGH RISK OF BIAS	6/8	NA	Participants dissimilar Limited measurement, puffs not counted	Indirectness of ENDS (device design no longer on the market)	No items noted
Polosa [33] SOME CONCERNS OF BIAS	9/10	NA	Participants dissimilar between exclusive ENDS users and dual users	ENDS type and quantity used not documented	No items noted
Polosa [32] SOME CONCERNS OF BIAS	9/10	NA	Participants dissimilar	No items noted	Data discrepancies in Table 2
Pulvers [38] Arnold [34] SOME CONCERNS OF BIAS	10/13	No participant blinding No treater blinding No assessor blinding	No other issues	Volunteer bias: high compensation with low-income participants	Spin: conclusion not supported by findings
Vardavas [35] HIGH RISK OF BIAS	8/10	NA	Dissimilar participants in comparisons Limited measurement, puffs not counted	Indirectness of ENDS (device type no longer on the market) No reporting of wash-out period	Spin: conclusion focus on results of one sub-group Spin: statistical significance interpreted as clinical relevance
Veldheer [36] SOME CONCERNS	12/13	No issues	Not all participants completed testing	One arm high nicotine ENDS Drop-outs not examined	Spin: previous study quoted as evidence for conclusion Power insufficient to support conclusions
Walele [37] HIGH RISK OF BIAS	6/9	NA	No control group Participants included persons with and without previous ENDS use Between group analyses mixed for completion and treatment compliance participants	Volunteer bias: recruitment from prior study, potential spill-over effect	Spin: Emphasis on secondary outcome Spin: effect modification on findings not supported

ENDS, electronic nicotine delivery system, e-cigarette; NA, not applicable; NS, nonsignificant finding

### Narrative synthesis: test findings

The test findings are for within-subject changes from baseline to final test measurement after ENDS substitution. Subgroup results are indicated in parentheses. Test measurements reaching statistical significance are reported in Additional file 1: Table S5 Statistically significant test measurements pre/post-test—acute studies and Additional file 1: Table S6 for follow-up studies.

#### Forced expiratory volume in 1 s (FEV_1_)

The FEV_1_ test measures the volume of air that is forced out of the lungs in 1 s; it can assess the severity and development of COPD [42, 43]. Twelve studies tested FEV_1_, five acute (one data not reported), and seven follow-up. Four acute tests were nonsignificant [27, 29, 30 (cigarettes), 30 (asthma)] and one acute study reported a significant decrease [23]. Three follow-up studies found no significant changes [25, 36, 38], four found statistically significant improvement [24, 26, 32, 33] and one reported a significant decrease [37].

#### Forced vital capacity (FVC)

FVC is the total volume of air that can be exhaled during a maximally forced expiration effort [44]. Twelve studies tested FVC within-subject. All five acute tests were nonsignificant [23, 27, 29, 30] (cigarettes), 30 (asthma)]. In the follow-up studies, three reported no significant effects [25, 36, 38], four found a significant increase [24, 26, 32, 33], and one [37] had a statistically significant decrease.

#### FEF_25–75_

The FEF_25–75_ is the average flow starting from the point at which 25% of the FVC has been exhaled to the point at which 75% has been exhaled [44]. It is potentially a sensitive marker of obstructive peripheral airflow [45]. Six studies conducted this test. One acute test was nonsignificant [27]. In the follow-up studies, three tests were nonsignificant [25, 36, 38], one indicated benefit [33] and one had a statistically significant decline [37].

#### Peak expiratory flow (PEF)

Peak expiratory flow (PEF) is the maximum airflow generated during a forceful exhalation, starting from full lung inflation [46]. Ten studies conducted this test. Four acute studies measured PEF; three tests had non-significant results [23, 27, 30] cigarettes] and a significant decline in two studies [29, 30] asthma]. Five follow-up studies showed no significant impact of ENDS use on PEF [24, 25, 28, 36, 38] and one had a significant decline [37].

#### FEV_1_/FVC%

The FEV_1_/FVC% is the percentage of the FVC expired in 1 s [44]. It is an indicator of obstructive defects, restrictive or mixed patterns of deteriorating lung function [47, 48]. Eight studies calculated this measurement. One acute study found a significant decrease [30] asthma] and three tests were not significant [27, 29, 30] cigarettes] and five follow-up studies had results that were not significant [24, 32, 33, 36, 38].

#### Impulse oscillometry (IOS)

The IOS test measures resistance to airflow and is more sensitive than spirometry for measuring peripheral airway disease [49]. Only the acute studies conducted this testing. Three studies found increased resistance with acute ENDS use [30, 31, 35], and in one study, test measurements were not significant after 30 min [22]. Three studies tested IOS on participants with asthma; two showed significant declines [30, 31] and one had no significant changes [22]. One study testing participants with COPD had nonsignificant test results [31].

#### Other lung function tests

Other tests were conducted in the acute studies. One test was total lung capacity, the volume of air in the lungs at maximal inflation [44]; one acute study [23] observed no significant changes in this test. Three tests conducted in two acute studies [23, 30] found no significant effects on Residual Volume, the volume of gas in the airways after maximal exhalation. One acute study [30] testing of Expiratory Reserve Volume, the volume of gas maximally exhaled after end-inspiratory tidal breathing [50] observed no significant changes for participants who smoked or for those with asthma.

#### Tabulation of testing findings

In summary, 66 test measurement findings were reported in the studies, out of which 43 (65%) were not significant. Significant findings were mixed, with 14 measuring declines and 9 showing improvement in lung function. A sensitivity analysis excluded the studies at high risk of bias, and the percentages were very similar for studies rated at some concerns (none industry-funded). None of the statistically significant test measurements was clinically relevant. See Additional file 1: Tables S5 and S6.

### Narrative synthesis: sub-group analyses

#### Dual use

Six studies evaluated differences in respiratory function between participants who exclusively used ENDS and those who used both ENDS and cigarettes (dual use). Four studies found no significant differences or improvements in those with dual use [25, 26, 34, 36]. Studies by Polosa et al. on asthma [33] and on COPD [32] observed that those who used ENDS exclusively had significant improvements in lung function tests FEV_1_, FVC and FEF_25–75_. While participants with dual use also showed improvements in these studies, the improvements were not as great compared to participants with exclusive ENDS.

#### Populations with underlying disease

Studies included participants with asthma, COPD, and serious mental illness.

Four follow-up studies were conducted with participants with various severities of asthma, with mixed findings. In these studies, participants with asthma with dual use showed improvement in all lung function tests, except the FVC test with exclusive ENDS use.

In one study [33], patients with mild to moderate asthma using ENDS on at least two consecutive follow-up visits over 24 months showed significant improvements in lung function tests FEV_1_, FVC, FEF_27–75_ for both exclusive and dual ENDS use, both groups of participants experiencing fewer exacerbations of asthma. Additional evidence from this study supported that ENDS substitution resulted in improvements: two patients who relapsed to smoking after ENDS use experienced worsening of their asthma outcomes. The study’s small sample size of 16 participants limits the confidence in these findings.

Three of four acute studies measuring IOS in participants with moderate asthma (*N* = 63) showed increased airway resistance with ENDS use [30, 31, 35], and one found no significant change [22]. These findings suggest possible airway irritation from ENDS use, but the test measurements in all three studies were not clinically relevant.

Only two studies were conducted with participants with COPD. A 5-year study of 39 patients with COPD [32] demonstrated significant improvements in participants with exclusive ENDS use aged 66.9 (± 5.8) that demonstrates that in older age, switching to ENDS may result in improvements in lung function over a longer period of time. In the other study [31], airway resistance in 16 patients with COPD after 10 min of ENDS use did not produce significant changes.

A cessation study [28] of patients with a serious mental illness found no clinically significant changes in their respiratory tests with ENDS substitution.

#### ENDS usage > 1 year

Three studies [32, 33, 37] with a longer duration of ENDS use indicated improved lung function in healthy participants and for those with an underlying health condition of COPD or asthma.

### GRADE

The certainty of the evidence was moderate to low in the acute studies and moderate to very low in the follow-up studies. Overall, the confidence in the evidence was rated as low. Ten RCTs and clinical trials were reduced to low confidence due to multiple risks of bias. Four RCTs and clinical trials were rated at some concerns, lowering their assessment to moderate. The two cohort studies were assessed as some concerns of bias, lowering their certainty to very low confidence. See Additional file 1: Table S7 for GRADE rating.

## Discussion

### Summary of main results

The 16 studies in this review conducted a total of 66 respiratory test measurements. No significant differences were reported in 43 tests (65%) between ENDS use and cigarette use. Nine follow-up studies found improvements in lung function tests. Declines in lung function tests were reported in 14 tests, 10 of which were from the acute studies, and all negative test results were from studies rated at high risk of bias. None of the statistically significant results indicated clinically relevant changes in lung functioning.

Findings on the respiratory health effects of ENDS substitution for smoking varied by health status and by the duration of ENDS use. For participants without respiratory disease, the acute studies did not show a clinically meaningful worsening of pulmonary function with ENDS use. Four acute and five short-term studies recorded no changes in healthy participants using ENDS. Also, one short-term study showed a decline in respiratory functioning in participants after they reverted from ENDS to cigarettes.

However, for participants with respiratory illnesses, the findings were mixed. For participants with asthma, two acute studies found a worsening of pulmonary function [30, 31], and one reported no significant change [22]. Yet these findings were not confirmed by a 24-month follow-up study [33] that observed no decline in respiratory functioning in participants with diagnosed asthma using ENDS and instead reported statistically significant improvement. Two studies were conducted with participants with COPD but the studies’ durations were diametrically different. A 5-year study showed significant test score improvements in patients with COPD who switched to ENDS [32] while an acute study reported no significant effects of ENDS use on COPD [31].

### Effect modification

A major problem with the findings is that the studies were not of sufficient duration. The beneficial effects of quitting cigarette use on lung function are not immediate and may take up to 2 years to manifest [51]. After stopping cigarette use, it takes 3 months for a reduction in the presence and severity of respiratory symptoms, 1 year for improvements in airway inflammation, and 8 years for improvements in lung diffusion capacity [52]. It is worth noting that improvements in spirometry testing can occur due to participants’ familiarity with the testing process rather than a clinically relevant improvement [53].

The duration of cessation is critical to accurately interpreting the results of the FEV_1_ test because improvements or lower rates of decline do not occur until after 1 year of cigarette abstinence [51, 52]. Nine studies conducted FEV_1_ tests, but with a duration of less than a year. Three studies conducted this test after at least 1 year of ENDS use and two reported statistically significant improvement in the test results [32, 33] and one found a significant decrease [37].

Improvement in symptoms after quitting cigarettes takes even longer for patients with respiratory diseases [54]. Lung function for COPD patients who stop smoking never improves; the loss of function is irreversible, and cessation can only help prevent further progression of the disease [51]. Evidence of the effects of ENDS substitution cannot be obtained from short-term studies if the duration does not account for recovery periods [55]. 

Another concern with the results of FEV_1_ tests is the age of the participants. The FEV_1_ test can measure improvements in those who stop cigarette use before age 30, but those who stop smoking after age 40 will show declines in FEV_1_ measurements that are not significantly different from those who continue to smoke [51]. Three studies had large age ranges in the participants. Seven studies included participants aged 30 (± 15), and two studies had participants aged 40 and above. Seven studies were conducted with participants 30 years old and younger. None of the authors accounted for the age of their participants as an effect modification of their findings for this spirometry test.

### Quality and bias assessments

One of the key observations of this review is the poor quality of much of the research literature, with ten of sixteen studies rated at high risk of bias and no studies rated at low risk of bias. Without discussing every item, we report below on the major areas of concern for biases in the research design, the conduct of the study, and reporting.

#### Study design

Blinding is a basic component of clinical research, where participants, clinicians, and researchers are prevented from knowing the intervention (or treatment); the participant receives in order to avoid the introduction of bias. With some studies, the blinding of participants is not possible because the difference appearances of ENDS and cigarettes is obvious, plus the lack of vapor with sham vaping is easy to identify. Yet it is possible to blind participants and clinicians to nicotine strengths or no nicotine, as was done by Veldheer et al. [36]. Researchers performing the statistical analyses can easily be blinded from identifying the intervention group of the individual participants. In five of the seven RCTs, blinding was not performed. See Table 2 JBI assessment and study biases.

Follow-up duration was a major limitation in study design. The seven acute studies per force and three follow-up studies [24, 26, 38] had a duration of less than 3 months. These studies have limited relevance for observing the potential effects of ENDS substitution because improvements in respiratory function take a minimum of 3 months to show benefit from cessation, and 2 years or more of cessation are needed for improvements in respiratory function (see Effect modification above).

#### Study conduct

A red flag in clinical research is unreported deviations from the study protocol (plan) because it may be an indication of the potential “cooking” of data to obtain desired or favorable results [56–58]. Two research teams did not indicate if they had a protocol [27, 29], and one had an unpublished protocol [24]. Four studies with protocols had discrepancies from the research design [25, 26, 28, 36], three of which we considered serious.

Another source of error in the findings is compliance bias—differences in subjects’ adherence to the planned treatment regimen or intervention [59]. Compliance bias was detected in six out of sixteen studies. Many participants continued to smoke even when they were instructed or “encouraged” not to [25, 37]. Four follow-up studies [24, 32, 33, 36] failed to report if the participants experienced adverse effects with ENDS use therefore it is not possible to determine if any of their participants curtailed ENDS use.

#### Reporting bias

Reporting bias is scientific misconduct. It happens from the selective reporting of results and excluding or concealing data [60–63]. Reporting bias also occurs when the study authors manoeuvre their discussion only to sources that conform to their desired conclusions [64, 65]. Reporting bias was detected in thirteen studies.

Most egregious, several studies published selective test results or did not provide actual pulmonary test measurements. Several authors characterized test results as “not significant” without reporting any data. Some figures in the articles included only *p* values (a probability statistic) or average differences between ENDS and cigarette user groups, but not the actual test measurement data.

In several studies, the authors manipulated their discussions or conclusions. In four studies [25, 27, 28, 32], the authors’ discussion presented the assumption that all other studies were in accord with their findings, that only one position exists (all’s well literature bias [66, 67]). In six studies [22–25, 30, 31], the authors offered only studies in support of their findings (one-sided reference bias [68]). Some authors unevenly highlighted one side of their study with the framing effect of language focused on the loss of health or risks [69–71].

As for conclusions, in eight studies, the conclusion was based on secondary endpoints (i.e. not the primary outcome) having no clinical significance. In the conclusions of five studies, there was an over-reliance on the statistical significance of *p* values [72] although the test results were not clinically relevant. See Table 2 JBI assessments and study biases.

### Comparison to other systematic reviews

Six systematic reviews published since 2020 have covered studies on the respiratory effects of ENDS, but their analysis does not match up with ours because of the very different types of studies they included. This makes comparing their conclusions with our conclusions untenable. The one partially comparable systematic review is the Larue et al. [73] meta-analysis based on 17 studies of acute respiratory outcomes from good-quality-rated cross-over studies and randomized parallel group studies. In accord with our findings, their meta-analysis did not find significant changes in spirometry tests with ENDS use.

Two systematic reviews conducted meta-analyses of cross-sectional studies (population surveys). Goniewicz et al. [74] examined two cross-sectional studies and one longitudinal population study on the respiratory effects of ENDS substitution. Their meta-analysis calculated ENDS substitution as producing a ~ 40% lower odds of negative respiratory outcomes of COPD, asthma, chronic bronchitis, emphysema, and wheezing. The meta-analysis of Chand and Hosseinzadeh [75] was comprised of 13 cross-sectional studies and calculated a significant association of between current e-cigarette use and asthma. Our review based on respiratory testing did not validate either the substantial benefit or the negative association with ENDS use. As is well established, cross-sectional studies are evidence of a possible association, but not causation.

Wills et al. [76] conducted a meta-analysis of human epidemiological studies. Eleven of the 15 asthma studies were with adolescent populations; they may not be indicative of outcomes for adults because adolescent asthma is known to go into remission in adulthood [77]. Their analysis incorporated studies of ENDS use on participants who had never smoked. Our PICO specified adults who smoke, so their conclusions are based on findings that do not match up with our study population.

Finally, the systematic reviews of Bozier et al. [78] and Bravo-Gutiérrez et al. [79] each anchored their conclusions in the evidence from in vitro (cell) and rat studies as well as including cross-sectional studies. We excluded in vitro studies because they “may not directly translate to adverse effects relevant to disease outcomes” in tobacco research [55]. As for animal tests on ENDS, almost all on rats, this study design does not reflect real life-use or human exposure levels as the rats’ exposure to ENDS is administered via intra-tracheally or nasally administered liquids or whole-body aerosol exposure [8]. Furthermore, respiratory studies on rodents have been dismissed by some researchers as not relevant to humans [80]. Because their analyses include non-human studies, these two systematic reviews are not comparable with ours.

### Recommendations for future research

Like other researchers of ENDS, our call is for longer-duration studies. Improved study quality is critical, requiring that research is conducted with an adequate number of participants. For statistical precision, future longitudinal studies should assess and stratify the results by smoking behaviour and history. Given the issues with treatment fidelity, exclusive ENDS use and dual use with cigarettes should be identified as separate categories. Reporting biases must be rooted out, either by the authors or by peer reviewers.

One concerning study design that should not be used is having ENDS users revert to smoking, as was done by Barna et al. [24]. This experimental design puts participants at risk for relapse to smoking.

### Limitations

Our systematic review has limitations, some derive from the quality of the studies themselves, and others from our conduct of the review. The studies have many limitations. The majority of studies were rated at high risk of bias, and no studies were at low risk. More than half of the studies, ten out of sixteen, were conducted with small sample sizes limiting their conclusions and precluding generalizability. In addition, acute effects contributed little to identifying health outcomes, nor did findings with significant *p* values indicate clinically relevant outcomes.

The review had limitations in its conduct. First, the quality and bias assessments were labor intensive, and the findings required more discussion than anticipated. Second, we had expected to find sufficient new studies published to continue this systematic review in the living mode (regular, ongoing updates), but this was not the case. We believe that our 100% compliance with PRISMA 2020 and AMSTAR2 requirements has served us well in conducting a rigorous and transparent systematic review with strong validity and reliability.

## Conclusions

Most of the studies showed no difference in respiratory parameters. Nearly two-thirds of the respiratory function tests found no significant effects of ENDS substitution for cigarette smoking. None of the statistically significant changes in test measurements was of clinical relevance. This indicates that ENDS substitution for smoking likely does not result in additional harm to respiratory health. Due to the high risk of bias and the small sample sizes in the majority of the studies, our certainty in this conclusion is low. Unfortunately, reporting spin is rampant, further eroding our confidence in the conclusions articulated by many of the study authors. To be able to inform policy and clinical practice, well done and robust studies are sorely needed to assess if ENDS substitution is a worthwhile harm reduction option for people who smoke.

### Supplementary Information

**Additional file 1**. Supplementary matertals.

## Data Availability

All data in this review were extracted from the original studies and available in the article. The data extraction forms and bias assessment reports are available in the Zenodo data repository https://zenodo.org/record/4835883#.YPnMqECxUuU.

## Abbreviations

COPD: Chronic obstructive pulmonary disease
ENDS: Electronic nicotine delivery system, e-cigarette
RCT: Randomized controlled trial
