# Correction to: Respiratory health efects of e-cigarette substitution for tobacco cigarettes: a systematic review

**DOI:** 10.1186/s12954-024-00968-1

**Published:** 2024-03-13

**Authors:** Maria Ahmed Qureshi, Robin W. M. Vernooij, Giusy Rita Maria La Rosa, Riccardo Polosa, Renee O’Leary

**Affiliations:** 1 Department of Clinical and Experimental Medicine, University of Catania, Catania, Italy; 2 Department of Nephrology and Hypertension, University Medical Center Utrecht and Julius Center for Health Sciences and Primary Care, Utrecht, Netherlands; 3 Centre of Excellence for the Acceleration of Harm Reduction, University of Catania, Via Santa Sofa, 89 Torre Biologica 11 Piano, 95123 Catania, Italy

**Correction to: Harm Reduction Journal (2023) 20:1**

10.1186/s12954-023-00877-9

Following publication of the original article [1], the authors pointed out that Springer Nature had made an error in its production of the manuscript with an uncorrected error in the final part of the Conclusions section. This originally appeared as:

“To be able to inform policy and clinical practice, well done and robust studies are sorely needed to assess. If ENDS substitution is a worthwhile harm reduction option for people who smoke.”

This has now been updated to:

“To be able to inform policy and clinical practice, well done and robust studies are sorely needed to assess if ENDS substitution is a worthwhile harm reduction option for people who smoke.”

The original article has been corrected.
