# Supplementary material for: Respiratory health effects of e-cigarette substitution for tobacco cigarettes: a systematic review
Source: Harm Reduct J. 2023 Oct 4;20:143. doi: 10.1186/s12954-023-00877-9 (PMC10552385; doi:10.1186/s12954-023-00877-9)

Supplemental Materials for Respiratory health effects of e-cigarette substitution for tobacco cigarettes: a systematic review, Qureshi AM, Vernooij RWM, La Rosa GRM, et al.

Contents

Table S1 PRISMA checklist

Table S2 Respiratory medical organizations grey literature search

Table S3 Quality rating rubric

Table S4 Excluded studies

Table S5 Statistically significant test measurements pre/post test – acute studies

Table S6 Statistically significant test measurements pre/post test – follow-up studies

Table S7 GRADE rating

Figure S1 Search syntax

Table S1 - PRISMA checklist


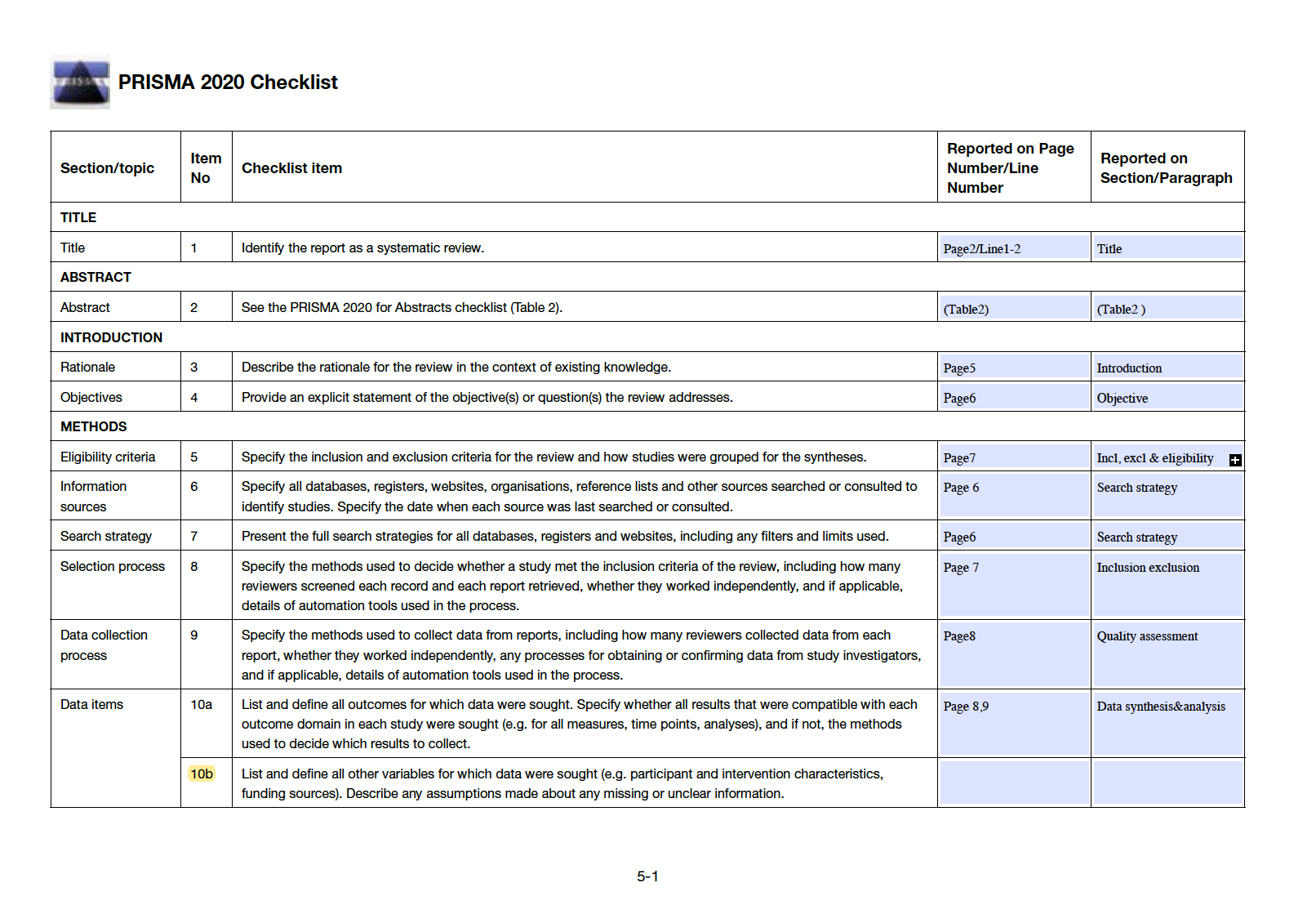


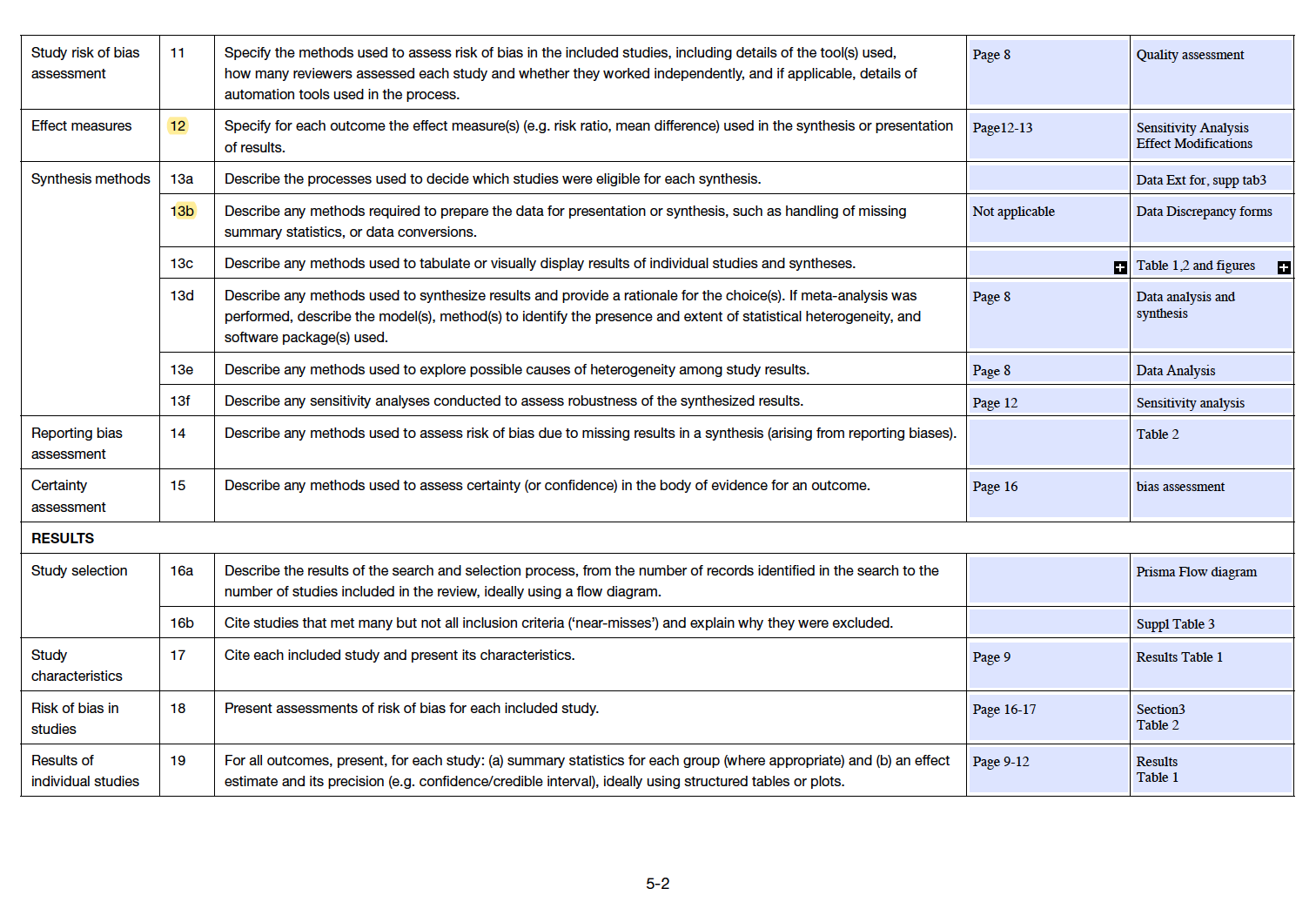


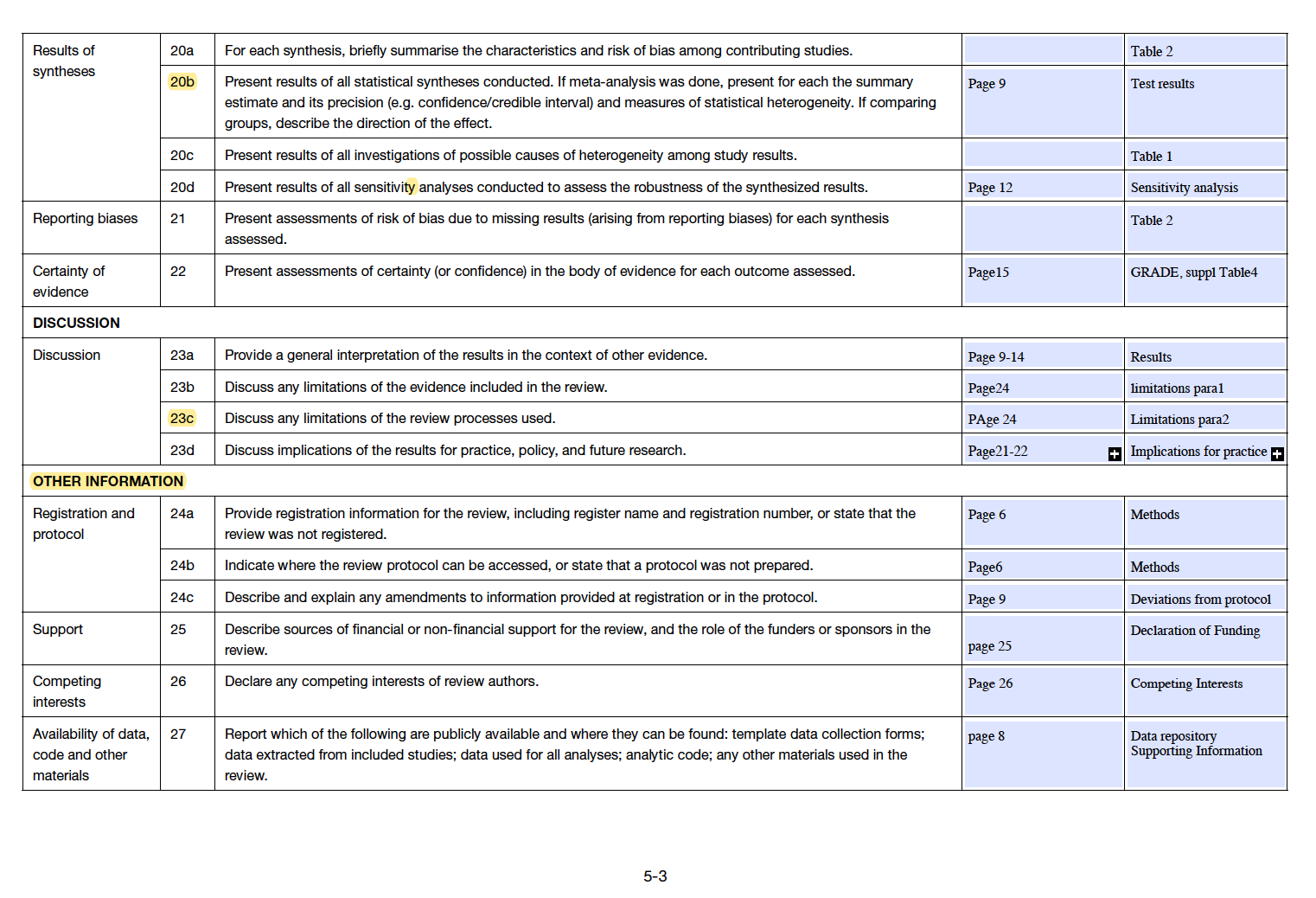


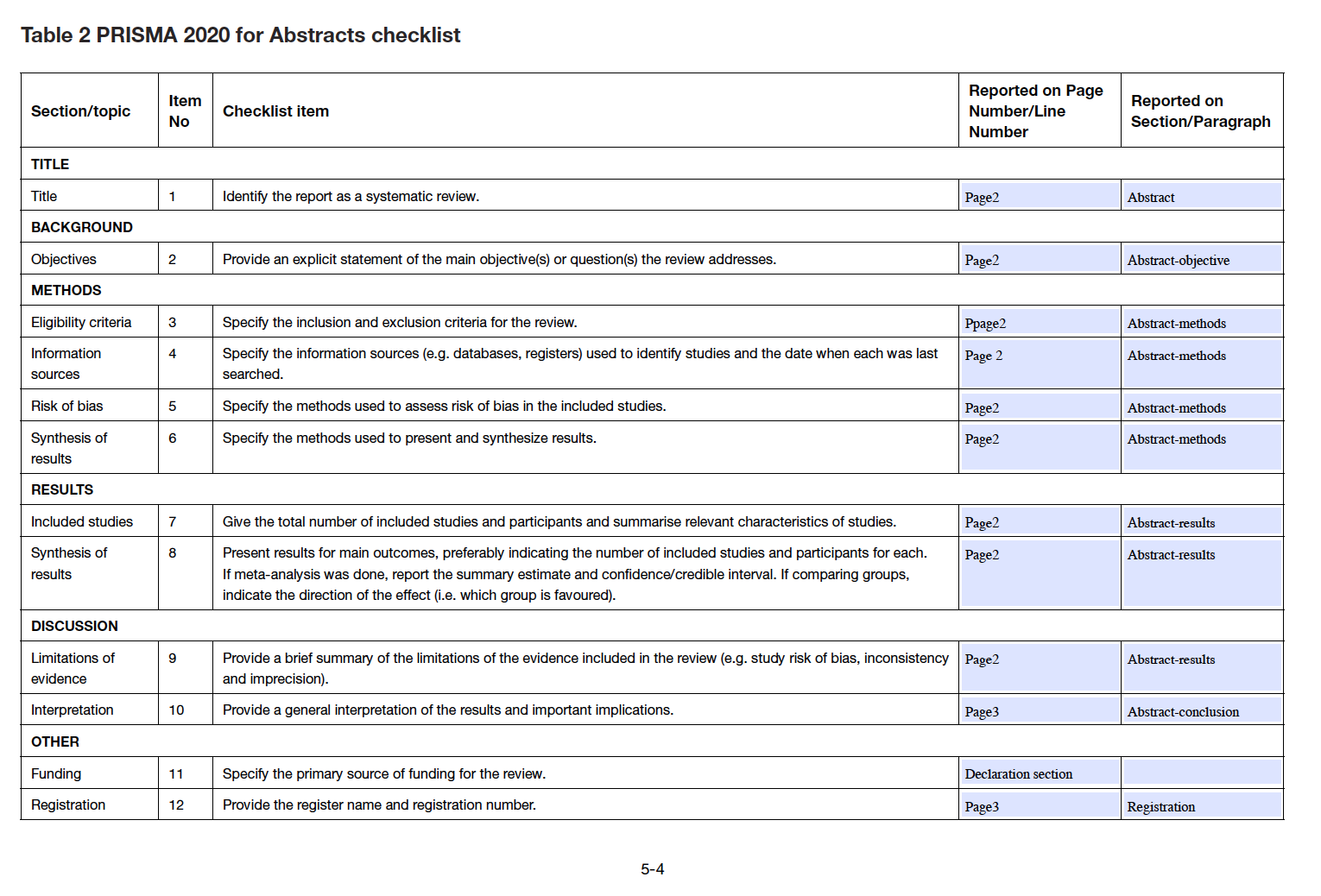


Table S2 – Respiratory medical organizations grey literature search

1. American Association for Respiratory Care
   [AARC.org](http://www.aarc.org/)
2. American College of Chest Physicians
   [chestnet.org](http://www.chestnet.org/)
3. American Lung Association

<https://www.lung.org/>

1. American Respiratory Care Foundation
   [ARCFoundation.org](https://www.arcfoundation.org/)
2. American Thoracic Society
   [thoracic.org](https://www.thoracic.org/)
3. Argentine Association of Respiratory Medicine

<https://www.aamr.org.ar/>

1. Asian Pacific Society of Respirology
   [apsresp.org](https://www.apsresp.org/about/contact.html)
2. Australian Lung Foundation
   [lungfoundation.com.au](https://lungfoundation.com.au/)
3. Australian & New Zealand Society of Respiratory Science
   [anzsrs.org.au](http://www.anzsrs.org.au/)
4. Bangladesh Lung Foundation
   [bdlungfoundation.com](http://www.bdlungfoundation.org/)
5. Brazilian Society of Pulmonology and Tisiology

<https://sbpt.org.br/portal/>

1. BREATHE

<https://www.lung.ca/>

1. British Thoracic Society
   [brit-thoracic.org.uk](http://www.brit-thoracic.org.uk/)
2. Chinese Association of Chest Physicians
   [cacpchina.org/](http://www.cacpchina.org/)
3. [Canadian Society of Respiratory Therapists](https://en.wikipedia.org/wiki/Canadian_Society_of_Respiratory_Therapists)

<https://www.csrt.com/>

1. Canadian Thoracic Society
   [lung.ca](http://www.lung.ca/)
2. Chinese Society of Respiratory Diseases
   [csrd.org.cn](http://www.csrd.org.cn/)
3. Chinese Thoracic Society
   [ctschina.org/cn/](http://www.ctschina.org/cn/)
4. European Respiratory Society
   [ersnet.org](https://www.ersnet.org/)
5. [European Society of Thoracic Surgeons](https://www.ests.org/)

<https://www.ests.org/>

1. Global Initiative for Asthma
   [ginasthma.com](http://www.ginasthma.com/)
2. Global Initiative for Obstructive Lung Disease
   [goldcopd.com](http://www.goldcopd.com/)
3. Ho Chi Minh City Respiratory Society
   [hoihohaptphcm.org](http://www.hoihohaptphcm.org/)
4. Hong Kong Lung Foundation
   [hklf.org](http://hklf.org/)
5. Hong Kong & Macau Chapter of the American College of Chest Physicians
   [fmshk.org](http://www.fmshk.org/accp/)
6. Hong Kong Tuberculosis, Chest & Heart Disease Association
   [antitb.org.hk](http://www.antitb.org.hk/en/)
7. Hong Kong Thoracic Society
   [hkts.hk](https://hkts.hk/)
8. Indian Chest Society
   [indianchestsociety.com/](http://www.indianchestsociety.com/)
9. International Primary Care Respiratory Group
   [theipcrg.org](http://www.theipcrg.org/)
10. Indonesian Society of Respirology
    [klikpdpi.com](http://www.klikpdpi.com/)
11. International Society for Respiratory Diseases
    [isrd.org](http://www.isrd.org/)
12. International Union Against Tuberculosis & Lung Disease
    [theunion.org](https://www.theunion.org/)
13. Japanese Respiratory Society
    [jrs.or.jp](https://www.jrs.or.jp/)
14. Korean Academy of Tuberculosis and Respiratory Disease
    [lungkorea.org/](https://www.lungkorea.org/)
15. Latin American Thoracic Association
    [alatorax.org](https://alatorax.org/)
16. Lung Foundation Australia
    [lungfoundation.com.au](https://lungfoundation.com.au/)
17. Malaysian Thoracic Society
    [mts.org.my](http://www.mts.org.my/)
18. Nigerian Thoracic Society

<http://nigerianthoracicsociety.org/>

1. Pan African Thoracic Society

<https://panafricanthoracic.org/>

1. Philippine College of Chest Physicians
   [philchest.org](http://philchest.org/)
2. Respiratory Care Indonesia
   [respina.org](http://respina.org/)
3. Sri Lanka College of Pulmonologists
   [copsl.lk](http://www.copsl.lk/)
4. Saudi Thoracic Society
   [saudithoracic.com](http://saudithoracic.com/)
5. Singapore Thoracic Society
   [thoracic.sg](https://www.thoracic.sg/)
6. Turkish Respiratory Society
   [solunum.org.tr](http://www.solunum.org.tr/eng/)
7. Thoracic Society of Australia & New Zealand
   [thoracic.org.au](https://www.thoracic.org.au/)
8. Taiwan Society of Pulmonary and Critical Care Medicine
   [tspccm.org.tw/](https://www.tspccm.org.tw/)
9. Thoracic Society of Thailand
   [thaithoracic.or.th](http://www.thaithoracic.or.th/)
10. Turkish Thoracic Society
    [toraks.org.tr/eng](https://www.toraks.org.tr/en/)
11. Vietnam Respiratory Society
    [hoihohapvietnam.org](https://hoihohapvietnam.org/)
12. World Association of Bronchology and Interventional Pulmonology
    [wabip.com](https://www.wabip.com/)
13. World Lung Foundation

<https://www.who.int/workforcealliance/members_partners/member_list/wlf/en/>

1. South African Thoracic Society
   [pulmonology.co.za](http://www.pulmonology.co.za/)

Table S3 – Quality rating rubric

| **Rating** | **JBI (except participant blinding)** | **Internal Validity** |
| --- | --- | --- |
| LOW | All yes | none |
| SOME CONCERNS | All yes | 1-2 |
| SOME CONCERNS | One no | 0-1 |
| HIGH | More than one no | any |
| HIGH | All yes | 3 or more |

Table S4 – Excluded studies

| No | Study | Reason for Exclusion |
| --- | --- | --- |
|  | Bowler, R. P., Hansel, N. N., Jacobson, S., et al. (2017). Electronic Cigarette Use in US Adults at Risk for or with COPD: Analysis from Two Observational Cohorts. *Journal of General Internal Medicine, 32*(12), 1315-1322. https://doi.org/10.1007/s11606-017-4150-7 | Study Design |
|  | Suhling, H., Welte, T., & Fuehner, T. (2020). Three patients with acute pulmonary damage following the use of E-cigarettes—A case series. *Deutsches Arzteblatt International, 117*(11), 177-182. https://doi.org/10.3238/arztebl.2020.0177 |  |
|  | Xie, W., H. Kathuria, P. Galiatsatos, M. J. Blaha, N. M. Hamburg, R. M. Robertson, A. Bhatnagar, E. J. Benjamin and A. C. Stokes (2020). "Association of Electronic Cigarette Use With Incident Respiratory Conditions Among US Adults From 2013 to 2018." JAMA Netw Open **3**(11): e2020816. |  |
|  | Xie, Z., D. J. Ossip, I. Rahman and D. Li (2020). "Use of Electronic Cigarettes and Self-Reported Chronic Obstructive Pulmonary Disease Diagnosis in Adults." Nicotine and Tobacco Research **22**(7): 1155-1161. |  |
|  | Antoniewicz, L., A. Brynedal, L. Hedman, M. Lundbäck and J. A. Bosson (2019). "Acute Effects of Electronic Cigarette Inhalation on the Vasculature and the Conducting Airways." Cardiovascular Toxicology **19**(5): 441-450. |  |
|  | Coppeta, L., Magrini, A., Pietroiusti, A., Perrone, S., & Grana, M. (2018). Effects of smoking electronic cigarettes on pulmonary function and environmental parameters. *Open Public Health Journal, 11*(1), 360-368. https://doi.org/10.2174/1874944501811010360 | No Comparator |
|  | Aherrera, A., A. Aravindakshan, S. Jarmul, P. Olmedo, R. Chen, J. E. Cohen, A. Navas-Acien and A. M. Rule (2020). "E-cigarette use behaviors and device characteristics of daily exclusive e-cigarette users in Maryland: Implications for product toxicity." Tob Induc Dis **18**: 93 |  |
|  | Kizhakke Puliyakote, A. S., A. R. Elliott, R. C. Sá, K. M. Anderson, L. E. Crotty Alexander and S. R. Hopkins (2020). "Vaping Disrupts Ventilation-Perfusion Matching in Asymptomatic Users." J Appl Physiol |  |
|  | Lee, S. M., R. Tenney, A. W. Wallace and M. Arjomandi (2018). "E-cigarettes versus nicotine patches for perioperative smoking cessation: a pilot randomized trial." PeerJ **6**: e5609 |  |
|  | Meo, S. A., M. A. et al (2019). "Electronic Cigarettes: Impact on Lung Function and Fractional Exhaled Nitric Oxide Among Healthy Adults." American Journal of Men's Health **13**(1). |  |
|  | Polosa, R., F. Cibella, P. Caponnetto, M. Maglia, U. Prosperini, C. Russo and D. Tashkin (2017). "Health impact of E-cigarettes: A prospective 3.5-year study of regular daily users who have never smoked." Scientific Reports **7**(1). |  |
|  | Ferrari, M., A. Zanasi, E. Nardi, A. M. Morselli Labate, P. Ceriana, A. Balestrino, L. Pisani, N. Corcione and S. Nava (2015). "Short-term effects of a nicotine-free e-cigarette compared to a traditional cigarette in smokers and non-smokers." BMC Pulmonary Medicine **15**(1). |  |
|  | Cobb, C. O., Foulds, J., Yen, M. S., et al. (2021, Apr 12). Effect of an electronic nicotine delivery system with 0, 8, or 36 mg/mL liquid nicotine versus a cigarette substitute on tobacco-related toxicant exposure: a four-arm, parallel-group, randomised, controlled trial. *Lancet Respir Med*. https://doi.org/10.1016/s2213-2600(21)00022-9 | No data |
|  | Rüther, T., Kahnert, K., Mader, M., et al. (2021). Reduction of bronchial response to mannitol after partial switch from conventional tobacco to electronic cigarette consumption. *Respiratory Medicine, 178*, Article 106324. https://doi.org/10.1016/j.rmed.2021.106324 |  |
|  | Caponnetto, P., D. Campagna, F. Cibella, et al (2013). "EffiCiency and Safety of an eLectronic cigAreTte (ECLAT) as tobacco cigarettes substitute: a prospective 12-month randomized control design study." PLoS One **8**(6): e66317. |  |
|  | Lucchiari, C., M. Masiero, K. Mazzocco, G. et al (2020). "Benefits of e-cigarettes in smoking reduction and in pulmonary health among chronic smokers undergoing a lung cancer screening program at 6 months." Addictive Behaviors |  |

Table S5 Statistically significant test measurements pre/post test – acute studies

| Study  Citation  Test | 23 | 27 | 29 | 30  Cigarettes | 30  Asthma | 22  Cig | 22  Asthma | 31  Cigarettes | 31  Asthma | 31  COPD | 35 |
| --- | --- | --- | --- | --- | --- | --- | --- | --- | --- | --- | --- |
| FEV_1_ | 4.4 (4.2-4.6) to 4.3 (3.9-4.6) | NS | NS | NS | NS |  |  |  |  |  |  |
| FEV_1_/FVC |  | NS | NS | NS | 75.19 (±8.23) to 74.58 (±7.96) |  |  |  |  |  |  |
| FVC | NS | NS | NS | NS | NS |  |  |  |  |  |  |
| PEF | NS | NS | 562 (62) to 531 (96) | NS | 7.58 (±2.02) to 7.12 (±2.08) |  |  |  |  |  |  |
| FEF_25-75_ |  | NS |  |  |  |  |  |  |  |  |  |
| IOS/  resistance |  |  |  | R5Hz 0.426(0.098) to 0.450(0.105)  R10Hz 0.382(0.096) to 0.402(0.098)  R20Hz 0.367(0.097) to 0.388(0.098) | Z5Hz 0.431 (0.121) to 0.464(0.149)  R10Hz 0.376(0.104) to 0.403(0.128)  R20Hz 0.362(0.101) to 0.386(0.114) | NS at end of test (30 min) | NS at end of test (30 min) | RAW  0.29 (0.12) to  0.31 (0.13)  sGaw  1.16 (0.47) to 1.03 (0.40) | RAW  0.38 (0.13) to 0.40 (0.11)  sGaw  NS | RAW  NS  sGaw  NS | Mean changes  Z5hz 0.376 to 0.409  R5 Hz 0.367 to 0.397  R10 Hz 0.325 to 0.353 |
| TLC | NS |  |  |  |  |  |  |  |  |  |  |
| RV | NS |  |  | NS | NS |  |  |  |  |  |  |
| ERV |  |  |  | NS | NS |  |  |  |  |  |  |

(Figures in parenthesis are the range of measurement test scores.)

Empty cell = test not conducted

Grey column – study at high risk of bias

ERV – expiratory reserve volume

FEV_1_ - forced expiratory volume, one second

FEV_1_/FVC - ratio of tests

FEV_25-75_ – forced expiratory volume capacity

FVC – forced vital capacity

IOS – impulse oscillometry system

min = minutes

NS = not significant

PEF – peak expiratory flow

R5Hz - airway resistance at 5 Hz

R10Hz - airway resistance at 10 Hz

R20Hz - air way resistance at 20 Hz

RAW - airways resistance IOS test

RV – residual volume

sGaw = specific conductance IOS test

TLC – total lung capacity

X5Hz - airway reactance at 5 Hz

Table S6 Statistically significant test measurements pre/post test –follow-up studies

| Study  citation  Test | 24 | 25 | 26 | 28 | 33 | 32 | 38/34 | 36 | 37 |
| --- | --- | --- | --- | --- | --- | --- | --- | --- | --- |
| FEV_1_ | ENDS test 0.004-0.312 higher than TC test measurement NR | NS | Arm 1 3.4 to 3.6  Arm 2  3.3 to 3.4  Arm 4  3.8 to 3.9  Arms 3 & 5 NS |  | 3.30 (0.78) to 3.40 (0.73) | 1.25 [0.98-1.78] to 1.42 [1.22. 1.95] | NS | NS | 3.506 (0.809) to 3.316 (0.784)^1^ |
| FEV_1_/FVC | NS |  |  |  | NS | NS | NS | NS |  |
| FVC | ENDS  test 0.018-0.256 higher than TC test measurement NR | NS | Arm 1 4.5 to 4.6  Arm 2 4.4 to 4.5  Arms 3-5 NS |  | 4.28 (0.90) to 4.43 (0.78) | 2.49 [2.08, 2.65] to 2.70 [2.17, 3.03] | NS | NS | 4.552 (1.031) to 4.369 (1.007)^1^ |
| PEF | NS | NS |  | NS |  |  | NS | NS | 509.34 (120.08) to 496.38 (108.16)^1^ |
| FEF_25-75_ |  | NS |  |  | 2.75 (0.72) to 3.11 (0.57) |  | NS | NS | 3.090 (1.082) to 2.835 (1.066)^1^ |

(Figures in parenthesis are the range of measurement test scores.)

Empty cell - test not conducted

Grey cell – study at high risk of bias

(standard deviation)

FEV_1_ - forced expiratory volume, one second

FEV_1_/FVC - ratio of tests

FEV_25-75_ – forced expiratory volume capacity

FVC – forced vital capacity

NR – not reported

NS = not significant

PEF – peak expiratory flow

^1^ reported as “decreases were not judged to be clinically significant”

Table S7 – GRADE ratings

| Study | Risk of bias rating | Design | Outcome | GRADE quality rating after assessment |
| --- | --- | --- | --- | --- |
| Acute - 7 |  |  |  |  |
| Chaumont | HIGH ROB | RCT | Within subject – 5 tests sig. decrease, 8 NS | LOW |
| Flouris | SOME ROB | Quasi | Within subject – 1 test decrease, 5 NS | MODERATE |
| Kerr | HIGH ROB | RCT | PEF reduced significantly post ENDS not TC | LOW |
| Kotoulas | HIGH  ROB | RCT | 2 test FEV1/FVC, PEF decrease in asthmatics,10 NS | LOW |
| Lappas | SOME  ROB | Quasi | 5 out of 8 IOS measurements significant in asthmatics | MODERATE |
| Palmidas | HIGH  ROB | Quasi | 1 test airway resistance decrease in ENDS users- 2 tests NS | LOW |
| Vardavas | HIGH ROB | Quasi | Inter and Intra group comparison, 1 test decrease in ENDS users. 5 tests NS | LOW |
| Follow-up - 9 | | | | |
| Barna | HIGH  ROB | Quasi | 1 test ENDS sig. less decline than TC, 3 NS | LOW |
| Cravo | HIGH  ROB | RCT | No clinical significance | LOW |
| D’Ruiz | HIGH  ROB | RCT | No significant differences between ENDS and TC users | LOW |
| Hickling | HIGH  ROB | Quasi | No clinical significance | LOW |
| Polosa asthma | SOME  ROB | Cohort | 3 tests improved in all users, 2NS, 3 in dual users, 2NS. | VERY LOW |
| Polosa COPD | SOME  ROB | Cohort | Improvement in ENDS group | VERY LOW |
| Pulvers/Arnold | SOME  ROB | RCT | No clinical significance | MODERATE |
| Veldheer | SOME  ROB | RCT | No clinical significance | MODERATE |
| Walele | HIGH ROB | RCT | No clinical significance | LOW |

Figure S1 Search syntax

2010-Jan 31, 2021, update on April 29, 2021.

(respiratory [Title/Abstract] OR lung [Title/Abstract] OR pulmonary [Title/Abstract]) AND (electronic cigarette [Title/Abstract] OR e-cigarette [Title/Abstract])

Screen shot May 18 2022


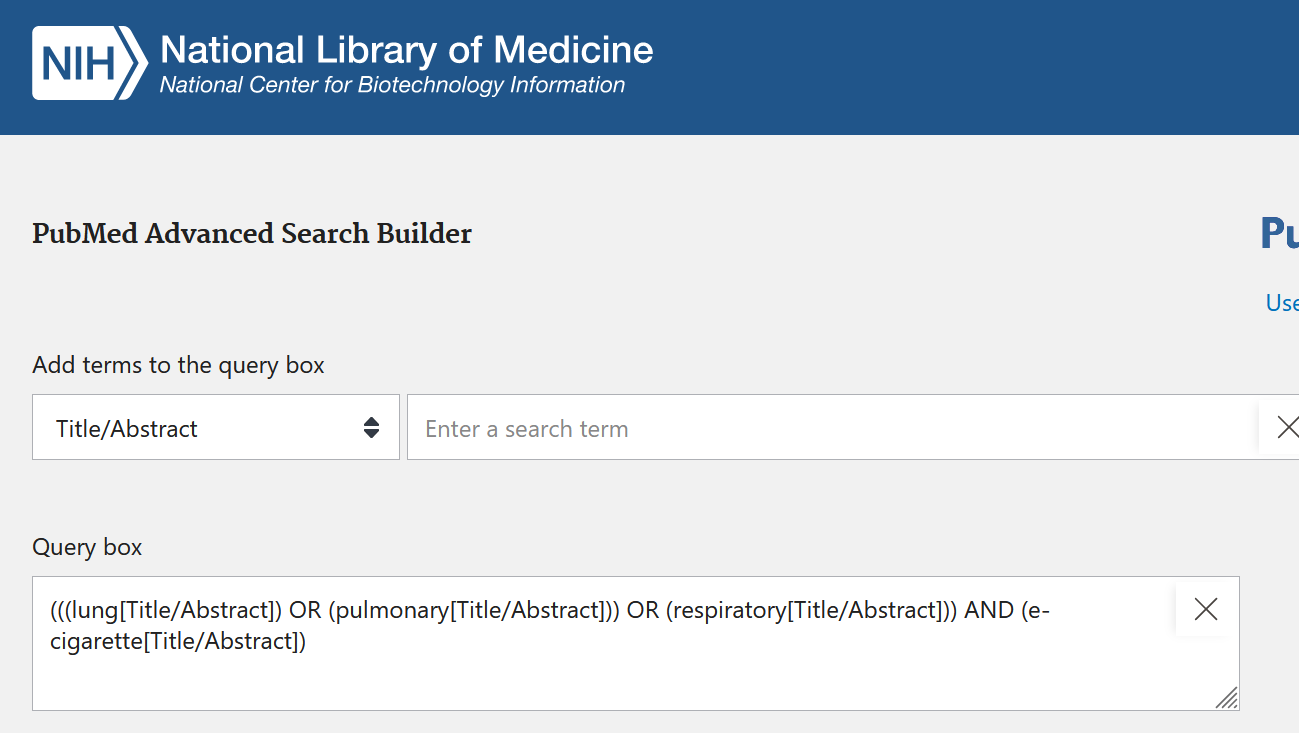

Supplement: Supplementary file 1 — Additional file 1. Supplementary matertals. [file 12954_2023_877_MOESM1_ESM.docx]
